# Might ART Adherence Estimates Be Improved by Combining Biomarker and Self-Report Data?

**DOI:** 10.1371/journal.pone.0167852

**Published:** 2016-12-14

**Authors:** Rebecca Rhead, Collen Masimirembwa, Graham Cooke, Albert Takaruza, Constance Nyamukapa, Cosmas Mutsimhi, Simon Gregson

**Affiliations:** 1 Imperial College London, Department of Infectious Disease Epidemiology, London, United Kingdom; 2 African Institute of Biomedical Research and Technology, Harare, Zimbabwe; 3 Imperial College London, Department of Medicine, London, United Kingdom; 4 Biomedical Research and Training Institute, Harare, Zimbabwe; Istituto di Genetica Molecolare, ITALY

## Abstract

**Background:**

As we endeavour to examine rates of viral suppression in PLHIV, reliable data on ART adherence are needed to distinguish between the respective contributions of poor adherence and treatment failure on high viral load. Self-reported data are susceptible to response bias and although biomarker data on drug presence and concentration can provide a superior, alternative method of measurement, complications due to drug-drug interactions and genetic variations can cause some inaccuracies. We investigate the feasibility of combining both biomarker and self-report data to produce a potentially more accurate measure of ART adherence.

**Methods:**

Data were taken from a large general-population survey in the Manicaland province, Zimbabwe, conducted in 2009–2011. HIV-infected adults who had initiated ART (N = 560) provided self-report data on adherence and dried blood spot samples that were analysed for traces of ART medication. A new three-category measure of ART adherence was constructed, based on biomarker data but using self-report data to adjust for cases with abnormally low and high drug concentrations due to possible drug-drug interactions and genetic factors, and was assessed for plausibility using survey data on socio-demographic correlates.

**Results:**

94.3% (528/560) and 92.7% (519/560) of the sample reported faithful adherence to their medication and had traces of ART medication, respectively. The combined measure estimated good evidence of ART adherence at 69% and excellent evidence of adherence at 53%. The regression analysis results showed plausible patterns of ART adherence by socio-demographic status with men and younger participants being more likely to adhere poorly to medication, and higher socio-economic status individuals and those living in more urban locations being more likely to adhere well.

**Conclusion:**

Biomarker and self-reported measures of adherence can be combined in a meaningful way to produce a potentially more accurate measure of ART adherence. Results indicate that ART adherence in Manicaland is at best 69%, which not only allows for considerable room for improvement but also suggests that the area may be falling short of the UNAIDS’ 90% target regarding viral suppression. Increased efforts are needed to improve ART adherence particularly amongst the young male population in rural areas of east Zimbabwe.

## Introduction

There is concern that people living with HIV (PLHIV) in sub-Saharan African, many of whom live in poverty and lack formal education, may have suboptimal adherence to HIV antiretroviral therapy (ART) [1–4]. Consistent adherence to ART is necessary to achieve viral suppression, prevent drug resistance, reduce transmission and delay disease progression [5,6]. Various structural and behavioural factors can lead to low adherence [7,8] which in turn can result in treatment failure. However, treatment failure can occur unrelatedly to adherence as a result of drug related problems such as drug resistance, poor absorption of medications, inadequate dosing, and drug-drug interactions. Therefore, reliable data on ART adherence are needed to distinguish between the respective contributions of poor adherence and treatment failure on high viral load.

UNAIDS recently set ambitious 90-90-90 targets for the HIV care cascade, with the objective that, by 2020, 90% of all PLHIV will be diagnosed; 90% of those diagnosed will receive sustained antiretroviral therapy (ART); and 90% of those on ART will have undetectable viral loads [9]. In order to achieve the last 90% target, it is important to not only identify those who continue to have detectable viral load despite receiving ART, but also to determine whether viral suppression has failed as a result of poor adherence, or other drug related problems. It is crucial to make this distinction as both causes of failed viral suppression require different solutions.

Self-reports of missed or irregular treatment doses have been used extensively in ART adherence research. They are inexpensive, easy to implement, and can identify patient-specific barriers to adherence [10]. However, self-reports are subject to bias with numerous studies suggesting that participants overstate actual adherence [11,12]. As a result, studies of ART adherence increasingly incorporate more objective measures into their research, electronic monitoring (MEMS) being the most common. MEMS caps fit standard-sized medication bottles and record the time and date of each opening as a presumptive dose. Data obtained from MEMS are often used in conjunction with self-reports to help determine the level of bias exhibited by the latter [11,13,14]. MEMS caps however are expensive ($80-$100 per cap) and thus often impractical, particularly in resource-limited settings. Such caps are also unable to measure whether the patient actually took pills out of the bottle when it was opened, if they took the correct dose, or if they consumed the pill that was taken out. Furthermore, MEMS caps are not compatible with patients using pillboxes or otherwise storing medications outside the capped bottles. Because of their cost, impracticality and inaccuracy, MEMS are particularly unsuitable for use in large-scale population-based surveys. Such surveys are necessary to obtain representative estimates of population-level adherence as well as risk factors for non-adherence among all PLHIV receiving ART.

A promising but comparatively less widely used objective measure of adherence is use of biological indicators. Detecting the presence and concentration of anti-retroviral drugs (ARVs) to determine if all medications are being taken as prescribed has been used successfully as a biomarker of drug adherence [15] and could be considered as a gold standard measure. Due to the development and validation of methods which use dried blood spot (DBS) as a sample matrix, and multiplex LC-MSMS methods that can detect more than one ARV at a time, this approach could be a useful one in measuring progress towards the third of the UNAIDS’ 90-90-90 targets. However, use of ARV biomarkers does have some limitations.

First, it may be necessary to test for a number of different ARVs to establish adherence. The majority of people living with HIV who are receiving ARVs in SSA do so under a national ART programme and take the same HAART combination. However, some patients will have unique combinations after failing the recommended first-line regimens. Whilst, some ART regimens are given as fixed dose combinations (FDCs) where detection of one component implies that all components are being taken, in other cases, patients receive separate pills so that ART detection must be expanded to include multiple components in their various combinations (of which there can be more than four). This can have major cost implications as there are no methods that can measure all possible combinations simultaneously.

Second, drug exposure can be affected by drug-drug interactions. Sub-therapeutic levels of NVP can potentially occur, not only from infrequent adherence but also from drug-drug interaction, most commonly with Rifampicin-based TB medication. Rifampicin is a potent inducer of CYP3A4 and an inducer of CYP2C9 [16–18]. NVP is particularly vulnerable to Rifampicin-based TB treatment [19]. Thai [20] and South African [21] investigators have found sub-therapeutic NVP concentrations in 21%-38% of co-infected patients on Rifampicin-based TB treatment taking standard NVP doses. Due to this drug-drug interaction, patients who are taking TB medication may be found to have sub-therapeutic concentrations of NVP despite adhering well to ART. In contrast, drugs such as Ketoconazole inhibit enzymes that metabolize some ARVs [22].

Third, though poor ART adherence can account for toxic concentration levels of (say) NVP as well as sub-therapeutic concentrations (since patients may take a large dose to compensate for previous or anticipated future missed doses), the genetic status of patients can also cause NVP concentrations to deviate from normal. NVP is principally metabolized by CYP3A4 and CYP2B6 [23,24] although CYP2D6 and CYP2C9 may also play a role [25]. A frequent CYP2B6 variant (516G→T) predicts decreased plasma clearance of NVP and increased plasma NVP exposure at steady state [26–28]. A less frequent CYP2B6 polymorphism, 983C→T, also predicts increased plasma NNRTIs exposure of a similar magnitude [29,30]. In short, both CYP2B6 516G→T and 983T→C are associated with increased steady-state plasma NVP exposure. Investigation of CYP2B6 516G→T polymorphism in various populations has shown that these alleles are largely absent in Caucasian populations [26,27], yet its frequency is as high as >34% in African-Americans and Ghanaians [31]. Both Mehlotra [32] and Dhoro [33] have also found slow metabolism variants of CYP2B6 and CYP3A4 to be particularly prevalent in west Africa. Patients who possess these alleles are still able to achieve viral suppression assuming they adhere to their ART. Similarly, a genetic variant of the enzyme CYP2B6 slows the activity of this enzyme, such that patients who possess it retain high levels of Efavirenz (EFZ). Having consistently high levels of EFZ, in turn, is associated with increased incidence of adverse drug reactions which can in turn increase the risk for poor adherence.

Fourth, results from tests that detect the presence of ART may be more difficult to interpret for some ARVs than for others. The presence of short half-life ARVs such as Lamivudine is more informative than the presence of long half-life ARVs such as EFZ and Nevirapine. This is because it is still possible to detect the presence of the latter several days after their intake, making poor adherence difficult to estimate. Furthermore, blood concentrations of nucleoside analogue inhibitors such as Lamivudine and Zidovudine do not reflect therapeutic concentrations since they are prodrugs; by contrast, the concentrations of non-nucleoside analogue inhibitors, such as EFZ and Nevirapine, do correlate with therapeutic effect. This disconnect between detection and concentration can complicate data interpretation.

In an attempt to address some of these difficulties in using either self-reports or biomarker data separately to measure ART adherence, we used data from a general population survey in east Zimbabwe to:

1. Measure ART adherence based on self-reported data and ARV drug detection;
2. Compare the overall estimates and examine the consistency of results on ART adherence obtained from these two different methods;
3. Construct a new measure of ART adherence which uses biomarker data in combination with self-report data where the former may be particularly unreliable; and
4. Assess the plausibility of this new combined measure by examining observed socio-demographic patterns of adherence.

## Data and Methods

Data for this study were taken from the 5^th^ wave of the Manicaland HIV/STD Prevention Project [34,35], a longitudinal survey which examines the dynamics of HIV infection and its impact in eastern Zimbabwe. The Manicaland project data collection team gathered data between October 2009 and July 2011 in 12 sites representing 4 socio-economic strata: small towns, agricultural estates, roadside trading centres, and subsistence farming villages (for more information visit *http://www.manicalandhivproject.org/data-collection-team.html*). Authors S.Gregson, C. Nyamukapa and A. Takaruza are affiliated with the Manicaland project and oversaw data collection during this time. In addition to completing a questionnaire, study participants also provided DBS, these were subject to HIV sero-testing and examined for the presence and concentrations of anti-retroviral drugs. Written informed consent was obtained from each study participant. Ethical approval for the Manicaland Project was obtained from the Medical Research Council of Zimbabwe and the Imperial College Research Ethics Committee.

DBS provide several advantages over conventional whole blood, plasma or serum sample collection [36–38]. The method is less invasive as only a small volume is required. DBS are taken from finger or heel prick as compared to conventional venous cannula. It is cheaper than collecting whole blood samples, requires simple storage and is easier to transfer as no freezers or dry ice are required. The dried blood matrix stabilizes many analyses and reduces the infection risk of HIV/AIDS and other infectious pathogens.

### Self-reported ART adherence

All survey participants provided information on their adherence to ART. Reportedly taking medication regularly regardless of whether they are feeling unwell or not, and not having stopped or forgotten to take this medication is seen as good evidence of self-reported adherence.

### Biomarker-based ART adherence

When the data were collected, the most common treatment for HIV was Stelanev (Nevirapine-NVP, Stavudine-d4T and Lamivudine-3TC)—an FDC. Those who did not tolerate this regimen or who were on co-treatment for TB were usually prescribed EFZ in place of NVP or were given combinations containing other drugs such as Zidovudine (AZT) as non-fixed dose combinations.

In this study, an LC-MSMS method for the detection of ARVs in DBS samples was developed according to the method of R ter Heine et al. [39] with minor modifications. DBS were punched out of a collection paper with a 0.25nm in diameter punch. The analytes were extracted from the punched-out disc using a mixture of acetonitrile, methanol and 0.2M zinc sulphate in water (1:1:2, v/v/v) containing the internal standard Ritonavir. Ritonavir was used as an internal standard as it is highly unlikely that patients would take ritonavir with either NVP or EFZ. 25 μL (microLitres) of the extract was injected onto the Waters Symmetry reversed-phase C18 column (50mm x 2.1mm-ID, 5μm) for separation from endogenous compounds. The analytes were quantified using a triple quadrupole mass spectrometer (API3000 Sciex). Due to poor peak resolution in some runs, some samples had ARVs determined either simultaneously or individually. The analytical run time was 3 mins with AZT, 3TC, NVP, EFZ and Ritonavir eluting out at 0.700, 0.763, 0.817, 0.615, 0.906 minutes respectively. All ARVs were analysed in the positive mode except EFZ which was analysed in negative mode in Multiple Reaction Monitoring mode (MRM). The target was to detect at least one ARV in each sample as a measure of adherence. In some samples, two or three ARVs were determined.

### New measure of ART adherence using biomarker data combined with self-report data

A three-category measure of ART adherence was produced based on drug presence and concentration of NVP. Poor adherence was defined as those with only one or no ARVs detected. Good evidence of adherence was defined as having non-therapeutic levels of NVP and at least one other ARV detected (non-therapeutic levels of NVP suggest that the participant is either not taking ART regularly or is taking an increased dose). Excellent adherence was defined as having therapeutic levels of NVP and at least one other ARV detected. In constructing this measure, the therapeutic range for NVP concentrations was taken to be 2–10 ug/ml, with concentrations below 2ug/ml treated as sub-therapeutic and those above 10ug/ml treated as potentially toxic.

Individuals with extremely high concentrations of NVP may be adhering well but possess slow metabolism variants of CYP2B6 and CYP3A4. To account for this, we constructed a new combined measure of ART adherence in which we assumed that those with highly toxic concentrations of NVP (>20 ug/ml), at least one other ARV detected, and self-reported good adherence had therapeutic levels of NVP. In addition, we assumed that participants with sub-therapeutic levels of NVP who reported taking TB medication and adhering well to ART were actually adhering to NVP but experiencing a drug-drug interaction.

### Social-demographic patterns of adherence

Demographic and socio-economic data from this survey were used to assess the plausibility of patterns of association between age, gender, socio-economic status, social capital, religion, marital status and area type and ART adherence in Manicaland using the new combined measure of adherence. Respondents socio-economic status (SES) was captured using a combined, four category measure of sellable and non-sellable assets created by Schur et al. [40]. Data on religion was categorised according to Manzou’s [41] grouping of Manicaland churches, where churches were grouped as ‘Christian’, ‘Spiritual’, ‘Traditional’ and ‘Other’. Finally, social capital was measured in the study as being the number of well-rated community groups that a respondent belonged to [42].

Bivariate analysis was conducted to test for associations between each of these socio-demographic variables and the combined measure of ART adherence. Then variables associated with adherence at p<0.1 were all included in a multivariable, multinomial logistic regression (poor adherence was used as the reference category).

## Results

### Self-reported adherence

Analysis was restricted to 607 survey participants who were diagnosed as HIV-positive in the study and answered ‘yes’ to the question, *Have you ever taken any drugs yourself that stop HIV causing AIDS*? 93.3% (n = 566/607) reported adhering to their ART regimen. These participants were also asked to specify whether they were taking ARVs or Cotrimoxazole. As shown in Table 1, 101 respondents reported taking Cotrimoxazole, 47 of whom had no trace of ARV medication in their blood sample. Cotrimoxazole can be taken prior to and/or during ART, which may have caused some confusion amongst respondents when answering this question. It is likely that the 54 respondents who reported taking Cotrimoxazole and had ART present in their blood sample were initiated onto Cotrimoxazole and had transitioned onto ART. The 47 respondents who reported taking Cotrimoxazole and had no trace of ART in their blood sample had likely yet to begin an ART regimen and, therefore, were excluded from the subsequent analysis.

**Table 1:** Number of participants taking ARV and Co-trimoxazole.

	ARV presence in blood sample		
What type of drugs are you taking?	None	> = 1	Total
ARV	39	448	487
Co-trimoxazole	47	54	101
Other	2	17	19
**Total**	**88**	**519**	**607**

After making this adjustment, 94.3% (n = 528/560) of the sample were reportedly adhering to their medication—they take this regularly (not just when they feel unwell), have never stopped for long periods, and do not occasionally forget a dosage.

### Biomarker-based ART adherence

No ARVs were detected in 7.3% (n = 41) of the sample. Only NVP was detected in 8.2% (n = 46), and only either AZT or 3TC (no NVP) was detected in 15.2% (n = 85). NVP and the presence of 3TC or AZT were detected in 69.3% of the sample (n = 388/560). Overall, results of DBS analysis showed that 92.7% (519/560) of the sample had at least one ARV present. EFZ was not found in any of the eligible blood samples; which was as expected since NVP was still widely prescribed in eastern Zimbabwe during the period of data collection.

Whilst the proportion adhering based on biomarkers was similar to that for self-reported adherence, we found no relationship between self-reported adherence and presence of ARV medication. As shown in Table 2, of the 32 participants who self-reported poor adherence, only 3 (9.4%) had no drugs present. Furthermore, 38 (7.2%) of those who reported good adherence were found to have no drugs present.

**Table 2:** Self-reported ART and ARV presence in DBS.

	No ARVs present			ARVs present			Total		
Self-reported ART adherence	n	col%	row%	n	col%	row%	n	col%	row%
Bad Adherence	3	7.3	9.4	29	5.6	90.6	32	5.7	100
Good Adherence	38	92.7	7.2	490	94.4	92.8	528	94.3	100
Total	41	100	7.3	519	100	92.7	560	100	100

### ART adherence using biomarker data combined with self-report data

**Fig 1:**
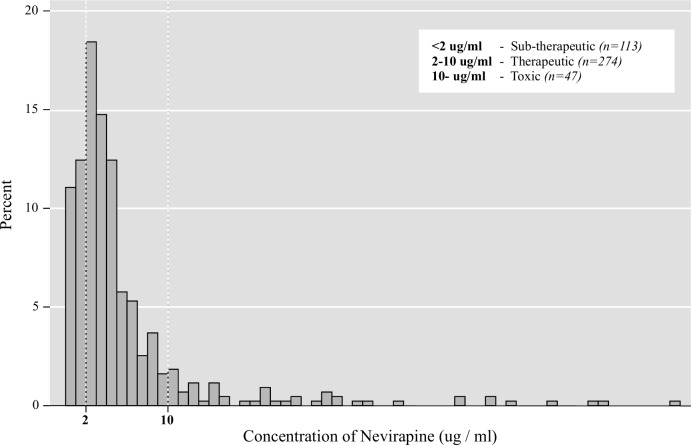
Distribution of concentrations of NVP in participants’ blood samples.

Excluding cases where NVP levels were found to be outside the normal therapeutic range but before accounting for the effects of possible genetic factors and drug-drug interactions, the combined measure of ART adherence yielded estimates of between 45% (good adherence) and 69% (excellent adherence) (as shown in Table 3). NVP was detected in 77.5% (434/560) of the DBS samples; of these, 63.1% (274/434) had therapeutic concentrations. The observed distribution of NVP concentrations is shown in Fig 1.

**Table 3:** New measure of adherence combining drug presence and NVP concentration while accounting for interaction with TB medication.

	Adherence (n = 560)	n	%	Std Er	95% CI	
No amendments	Poor	172	30.7%	0.02	0.27	0.35
	Good	133	23.8%	0.02	0.20	0.27
	Excellent	255	45.5%	0.02	0.41	0.50
Amended to account for TB medication and genetic factors	Poor	172	30.7%	0.02	0.27	0.35
	Good	90	16.1%	0.02	0.13	0.19
	Excellent	298	53.2%	0.02	0.49	0.57

We then created an amended measure of adherence by identifying participants who were potentially adhering well to their medication, but had non-therapeutic concentrations of NVP as a result of an interaction with TB medication, or a genetic defect. In our investigation to identify the latter, we found 47 participants had toxic levels of NVP (>10ug/ml), of whom, 20 had extremely high concentrations (>20 ug/ml), likely a result of poor drug absorption. 18 of these 20 participants had at least one other ARV detected and self-reported good adherence, suggesting that, aside from having unusually high levels of NVP, they were adhering to their ART. For our amended measure therefore, these 18 participants were assumed to be experiencing poor drug absorption as a result of genetic defects and were reclassified from good to excellent ART adherence. In our investigation to identify potential drug-drug interactions, we found 25 participants with sub-therapeutic levels of NVP who reported adhering well and taking TB medication; these cases were also reclassified from good to excellent ART adherence.

Following these adjustments, the amended measure of adherence (Table 3), combining data on drug presence and concentration with self-reported data, yielded estimates of adherence between 53% (good adherence) and 69% (excellent adherence).

### Socio-demographic patterns of adherence based on the combined biomarker and self-report measure of adherence

**Fig 2:**
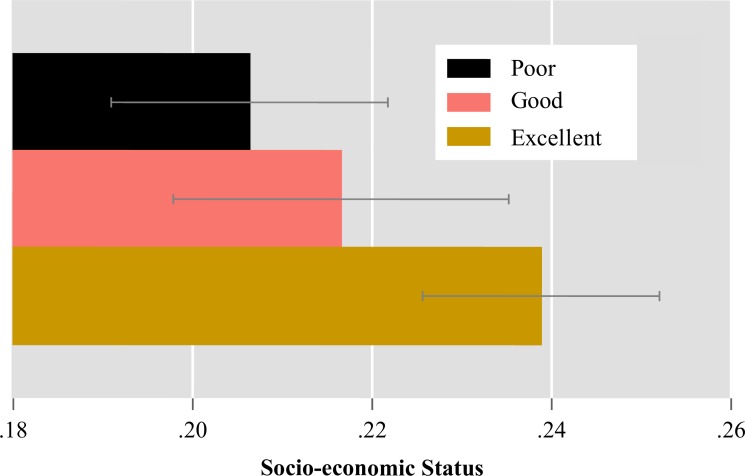
Mean (and 95%CI) socio-economic status scores by ART adherence group.

Using the combined measure of adherence that attempts to account for biomarker inaccuracies, in a bivariate analysis, we found that better adherence was associated with older ages, female gender, Christian religion, residence in an agricultural estate or in or close to an urban/peri-urban area, and higher SES (Table 4). Those who adhered well had a higher proportion of participants who belonged to the top two SES quintiles than those with poor adherence (Fig 2).

**Table 4:** Patterns of association between socio-demographic characteristics and a combined measure of ART adherence (bivariate analysis).

	Level of adherence						Total		
	Poor		Good		Excellent				
Covariate	No.	Row %	No.	Row %	No.	Row %	No.	Row %	p†
Age									
15–29	20	46.5	8	18.6	15	34.9	43	100	0.037
30–39	71	34.5	35	17	100	48.5	206	100	
40–49	55	27.8	28	14.1	115	58.1	198	100	
50–65	26	23	19	16.8	68	60.2	113	100	
Total	172	30.7	90	16.1	298	53.2	560	100	
Gender									
Male	45	37.8	10	8.4	64	53.8	119	100	0.018
Female	127	28.8	80	18.1	234	53.1	441	100	
Total	172	30.7	90	16.1	298	53.2	560	100	
Marital Status									
Widowed	56	25.9	37	17.1	123	56.9	216	100	0.335
Divorced/separated	20	30.8	12	18.5	33	50.8	65	100	
Still in union	92	34.6	39	14.7	135	50.8	266	100	
Total	168	30.7	88	16.1	291	53.2	547	100	
Social Capital									
No group	44	31.2	23	16.3	74	52.5	141	100	0.980
= >1 group	128	30.5	67	16	224	53.5	419	100	
Total	172	30.7	90	16.1	298	53.2	560	100	
Member of church group?									
Yes	136	29.1	76	16.2	256	54.7	468	100	0.227
No	31	38.3	12	14.8	38	46.9	81	100	
Total	167	30.4	88	16	294	53.6	549	100	
Employment									
Employed	54	29.5	24	13.1	105	57.4	183	100	0.288
Unemployed	118	31.3	66	17.5	193	51.2	377	100	
Total	172	30.7	90	16.1	298	53.2	560	100	
Education									
Primary or less	70	27.2	50	19.5	137	53.3	257	100	0.072
Secondary or higher	102	33.7	40	13.2	161	53.1	303	100	
Total	172	30.7	90	16.1	298	53.2	560	100	
SES									
0 (Low)	22	32.8	21	31.3	24	35.8	67	100	0.003
1 (Middle—low)	66	33.3	26	13.1	106	53.5	198	100	
2 (Middle—high)	45	30.4	22	14.9	81	54.7	148	100	
3 (High)	39	27.1	21	14.6	84	58.3	144	100	
Total	172	30.9	90	16.2	295	53	557	100	
Religion									
Christian	83	28.6	36	12.4	171	59	290	100	0.014
Traditional/Other	26	31.7	14	17.1	42	51.2	82	100	
Spiritual	55	33.7	37	22.7	71	43.6	163	100	
Total	164	30.7	87	16.3	284	53.1	535	100	
Area Type									
Subsistence farming villages	86	39.1	46	20.9	88	40	220	100	<0.001
Roadside settlements	33	30.8	15	14	59	55.1	107	100	
Tea / forestry estates	19	17.6	19	17.6	70	64.8	108	100	
Towns	34	27.2	10	8	81	64.8	125	100	
Total	172	30.7	90	16.1	298	53.2	560	100	
Urbaness (Distance from nearest growth point)									
<5 km	40	28.2	19	13.4	83	58.5	142	100	0.052
5–19 km	29	22.3	19	14.6	82	63.1	130	100	
20–30 km	53	35.8	26	17.6	69	46.6	148	100	
>30 km	48	34.8	26	18.8	64	46.4	138	100	
Total	170	30.5	90	16.1	298	53.4	558	100	

†chi2 p value for associated between covariate and level of adherence

Variables with p < .1 will be included in multivariable analysis

Multinomial regression (Table 5) confirmed that, while accounting for all socio-demographic factors simultaneously, older participants were more likely have excellent adherence than younger patients aged 15–29 (30–39 years: OR = 2.18; 95% CI: 0.97–4.88; 40–49 years: OR = 2.98; 95% CI: 1.30–6.83; 50–65 years: OR = 4.39; 95% CI: 1.67–11.51). Women are also significantly more likely to have good and excellent levels of adherence than men (good: OR = 3.47; 95% CI: 1.53–7.86; excellent: OR = 1.85; 95% CI: 1.11–3.10). Those who belonged to spiritual church denominations (Marange Apostolic, Zviratidzo Apostolic, Other Apostolic, Zionist and Mughodi church denominations.) were less likely to have excellent adherence compared to those who attend Christian churches (OR = 0.59; 95% CI: 0.36–0.95). Finally, residents of towns and roadside settlements were more likely to have excellent adherence compared to those in rural villages (towns: OR = 2.12; 95% CI: 1.08–4.13; roadside settlement: OR = 1.90; 95% CI: 1.05–3.42), and people on ART in tea estates were more likely to have both good and excellent adherence than those in villages (good: OR = 2.47; 95% CI: 1.09–5.59; excellent: OR = 3.94; 95% CI: 2.03–7.63).

**Table 5:** Patterns of association between socio-demographic characteristics and a combined measure of ART adherence (multinomial multivariate logistic regression).

ART Adherence (Poor adherence is base)	RRR	Std.	z	P	95% Conf.	
**Excellent Adherence**						
Age						
15–29	-	-	-	-	-	-
30–39	2.18	0.90	1.89	0.06	0.97	4.88
40–49	2.98	1.26	2.58	0.01	1.30	6.83
50–65	4.39	2.16	3.01	0.00	1.67	11.51
Gender						
Male	-	-	-	-	-	-
Female	1.85	0.49	2.36	0.02	1.11	3.10
Religion						
Christian	-	-	-	-	-	-
Traditional/Other	0.73	0.22	-1.02	0.31	0.40	1.34
Spiritual	0.59	0.14	-2.17	0.03	0.36	0.95
SES						
0 (Low)	-	-	-	-	-	-
1 (Middle—low)	1.07	0.40	0.18	0.86	0.52	2.22
2 (Middle—high)	1.22	0.47	0.51	0.61	0.57	2.61
3 (High)	1.15	0.47	0.35	0.72	0.52	2.56
Education						
Primary or less	-	-	-	-	-	-
Secondary or higher	1.10	0.28	0.39	0.70	0.67	1.81
Site Type						
Subsistence Farming Villages	-	-	-	-	-	-
Roadside Settlement	1.90	0.57	2.13	0.03	1.05	3.42
Tea / Forestry Estate	3.94	1.33	4.07	0.00	2.03	7.63
Town	2.12	0.72	2.20	0.03	1.08	4.13
Urbanness						
<5 km	-	-	-	-	-	-
5–19 km	1.44	0.47	1.12	0.26	0.76	2.74
20–30 km	0.81	0.27	-0.62	0.54	0.42	1.57
>30 km	0.71	0.24	-1.02	0.31	0.37	1.37
Cons	0.27	0.18	-1.92	0.06	0.07	1.03
**Good Adherence**						
Age						
15–29	-	-	-	-	-	-
30–39	1.45	0.75	0.72	0.47	0.53	3.98
40–49	1.18	0.65	0.31	0.76	0.41	3.45
50–65	1.81	1.12	0.96	0.34	0.54	6.09
Gender						
Male	-	-	-	-	-	-
Female	3.47	1.45	2.97	0.00	1.53	7.86
Religion						
Christian	-	-	-	-	-	-
Traditional/Other	1.33	0.55	0.69	0.49	0.59	3.00
Spiritual	1.63	0.51	1.56	0.12	0.88	3.00
SES						
0 (Low)	-	-	-	-	-	-
1 (Middle—low)	0.34	0.14	-2.60	0.01	0.15	0.77
2 (Middle—high)	0.46	0.20	-1.75	0.08	0.20	1.10
3 (High)	0.52	0.24	-1.41	0.16	0.21	1.29
Education						
Primary or less	-	-	-	-	-	-
Secondary or higher	0.75	0.25	-0.88	0.38	0.39	1.43
Site Type						
Subsistence farming villages	-	-	-	-	-	-
Roadside settlements	1.02	0.42	0.06	0.95	0.46	2.27
Tea / forestry estates	2.47	1.03	2.17	0.03	1.09	5.59
Town	0.38	0.20	-1.83	0.07	0.14	1.07
Urbanness						
<5 km	-	-	-	-	-	-
5–19 km	1.15	0.53	0.30	0.77	0.47	2.82
20–30 km	0.67	0.31	-0.88	0.38	0.27	1.64
>30 km	0.80	0.36	-0.50	0.62	0.33	1.94
Cons	0.31	0.27	-1.35	0.18	0.06	1.71

We replicated the analysis in Table 5 using the un-amended biomarker measure of adherence to compare results obtained from both measures of adherence. In our examination of these findings we find no significant changes to the patterns of association, likely due to the relatively small overall sample size and number of re-classifications.

## Discussion

The biomarker data obtained for this study were assessed for their ability to provide an accurate alternative to self-reported adherence. Although certainly more accurate than self-reported adherence, biomarker data indicating drug presence and concentrations within DBS samples are susceptible to the effects of drug-drug interactions and host genetic defects.

Upon examination of the data, self-reported adherence was found to be extremely high, as was the proportion of the sample who had detectable ARVs. However, no relationship was found between those who had no detectable ARVs and those who reported poor adherence. A three-category measure of ART adherence was then produced based on ARVs detected and NVP concentration. This estimated adherence at 45%-69%. We also tentatively proposed a means of incorporating self-reported data with the biomarker data to avoid misclassifying people who adhere to their regimen but are unable to adequately metabolize ARVs. In doing this, 25 people were identified as having potential drug-drug with their TB medications, and 18 were identified as having host genetic defects. Accounting for these issues produced a potentially more accurate measure which estimated adherence at between 53% and 69%, suggesting that self-reported data, and to a lesser extent biomarker data, may have overestimated adherence. The finding that adherence has been over reported is in line with previous studies which indicate that self-reported adherence is heavily biased.

Results of bivariate and multinomial multivariate logistic regression analyses showed that men, younger participants, and participants with lower SES were more likely to adhere poorly to their ART medication. Results also indicated that church denomination and area type were significantly related to adherence. Apostolic churches stress faith healing and discourage medical treatments so it is plausible that members would have poorer adherence. Residents of farming and rural villages were less likely to adhere well to their ART medication than those in more urban areas. This could be because those in urban areas, particularly towns, have better access to health services [43]. Additionally, if residents of tea/forestry estates are too ill to work, they may be at risk of losing their job, and with it, their home. This may motivate such residents to adhere well to their ART regimen. These findings are highly plausible, providing initial support for the validity of our combined measure, although further validation is required to confirm its utility.

One limitation of this study is that the combined measure of adherence still relies on self-report data to an extent. However, the most important self-report data used (on taking TB medication) may not suffer from less bias than self-reported ART adherence. We also acknowledge the lack of certainty regarding the amendments made to our measure of adherence which account for drug interactions and genetic factors (particularly the latter). The extent to which the sample exhibits genetic abnormalities which interfere with the metabolism of EFZ and NVP ideally would be confirmed through further genetic testing that we were not able to conduct in this study.

In a sense, these findings are outdated, as NVP has largely been replaced by EFV in current regimens, however, these findings are relevant to a wide range of possible treatments. For example, EFZ can also be affected by similar genetic factors and is also vulnerable to drug-drug interactions (particularly treatment for hepatitis C), and, as such, could by accounted for in the same way this study has done with NVP and TB medication.

Finally, we found that, for some participants, there was confusion over whether they were receiving ART, Cotrimoxazole, or both. Patients in Manicaland may not be given enough information regarding their ARVs and, as a result, may be unable to make the distinction between ART and Cotrimoxazole. At the time these data were collected, Cotrimoxazole was given to those diagnosed but ineligible for ART according to WHO clinical staging. Some were later given ART in addition to Cotrimoxazole (for opportunistic infections), or transitioned onto ART when they become eligible, possibly with little to no explanation. Particularly for residents with poor access to further information regarding their regimen, Cotrimoxazole may have become synonymous with ART treatment in Manicaland. Therefore, it is important that health personnel fully inform their patients about their medication and specifically why they are being put onto Cotrimoxazole and/or ART after testing positive. This may help to reduce patient confusion and improve future efforts to measure ART adherence.

We acknowledge that of the 18 people who were reclassified under the assumption that their high levels of NVP were a result of genetic defect, some may simply have been taking too much of their medication. However, given the unusually high levels of NVP, and the prevalence of genetic factors amongst the population we propose that those with highly toxic concentrations of NVP (>20 ug/ml), at least one other ARV detected, and self-reported good adherence are potentially adhering well but are unable to properly metabolise NVP. This can potentially be confirmed with the use of MEMS in future studies. In the introduction of this paper, MEMS was deemed to be an unsuitable method of measuring adherence in large-scale general population surveys, mostly for reasons of cost. However, MEMS could be used to measure adherence in smaller subgroups identified using our proposed combined measure of adherence. Measuring adherence levels in this subgroup with MEMS could help to determine the likelihood of genetic factors interfering with drug metabolism.

Overall, this research demonstrates that biomarker and self-reported measures of adherence can be combined in a meaningful way to produce a potentially more accurate measure of ART adherence. Results of this analysis indicate that Manicaland, in the period the study was conducted, good evidence of adherence estimated at between 54% and 69%. Increased efforts are needed to improve ART adherence particularly amongst the young male population in rural areas of east Zimbabwe, if the area is to meet these UNAIDS’ targets by 2020.

## References

[1] Harries AD, Nyangulu DS, Hargreaves NJ, Kaluwa O, Salaniponi FM. Preventing antiretroviral anarchy in sub-Saharan Africa. Lancet Lond Engl. 2001;358: 410–414. 10.1016/s0140-6736(01)05551-9 11502341

[2] Liechty CA, Bangsberg DR. Doubts about DOT: antiretroviral therapy for resource-poor countries. AIDS Lond Engl. 2003;17: 1383–1387. 10.1097/00002030-200306130-00013 12799560

[3] Pearson CR, Simoni JM, Hoff P, Kurth AE, Martin DP. Assessing antiretroviral adherence via electronic drug monitoring and self-report: an examination of key methodological issues. AIDS Behav. 2007;11: 161–173. 10.1007/s10461-006-9133-3 16804749 PMC5096443

[4] Fox MP, Rosen S. Patient retention in antiretroviral therapy programs up to three years on treatment in sub-Saharan Africa, 2007–2009: systematic review. Trop Med Int Health TM IH. 2010;15 Suppl 1: 1–15. 10.1111/j.1365-3156.2010.02508.x PMC2948795 20586956

[5] Vanhove GF, Schapiro JM, Winters MA, Merigan TC, Blaschke TF. Patient compliance and drug failure in protease inhibitor monotherapy. JAMA. 1996;276: 1955–1956. 8971062

[6] Montaner JS, Reiss P, Cooper D, Vella S, Harris M, Conway B, et al A randomized, double-blind trial comparing combinations of nevirapine, didanosine, and zidovudine for HIV-infected patients: the INCAS Trial. Italy, The Netherlands, Canada and Australia Study. JAMA. 1998;279: 930–937. 9544767 10.1001/jama.279.12.930

[7] Ware NC, Idoko J, Kaaya S, Biraro IA, Wyatt MA, Agbaji O, et al Explaining Adherence Success in Sub-Saharan Africa: An Ethnographic Study. PLOS Med. 2009;6: e1000011. 10.1371/journal.pmed.1000011 PMC2631046 19175285

[8] Ajose O, Mookerjee S, Mills EJ, Boulle A, Ford N. Treatment outcomes of patients on second-line antiretroviral therapy in resource-limited settings: a systematic review and meta-analysis. AIDS Lond Engl. 2012;26: 929–938. 10.1097/QAD.0b013e328351f5b2 22313953

[9] HIV/AIDS JUNPo. 90–90–90—An ambitious treatment target to help end the AIDS epidemic | UNAIDS [Internet]. 2014. Available: http://www.unaids.org/en/resources/documents/2014/90-90-90

[10] Mills EJ, Nachega JB, Buchan I, Orbinski J, Attaran A, Singh S, et al Adherence to antiretroviral therapy in sub-Saharan Africa and North America: a meta-analysis. JAMA. 2006;296: 679–690. 10.1001/jama.296.6.679 16896111

[11] Km M, Sm G, Sl B, C W-L, S C, A F. Medication adherence in patients with HIV infection: a comparison of two measurement methods. AIDS Read. 1999;9: 329–338. 12737122

[12] Simoni JM, Huh D, Wang Y, Wilson IB, Reynolds NR, Remien RH, et al The Validity of Self-Reported Medication Adherence as an Outcome in Clinical Trials of Adherence-Promotion Interventions: Findings from the MACH14 Study. AIDS Behav. 2014;18: 2285–2290. 10.1007/s10461-014-0905-x 25280447 PMC4495040

[13] Golin CE, Liu H, Hays RD, Miller LG, Beck CK, Ickovics J, et al A prospective study of predictors of adherence to combination antiretroviral medication. J Gen Intern Med. 2002;17: 756–765. 10.1046/j.1525-1497.2002.11214.x 12390551 PMC1495120

[14] Arnsten JH, Demas PA, Farzadegan H, Grant RW, Gourevitch MN, Chang C-J, et al Antiretroviral Therapy Adherence and Viral Suppression in HIV-Infected Drug Users: Comparison of Self-Report and Electronic Monitoring. Clin Infect Dis Off Publ Infect Dis Soc Am. 2001;33: 1417–1423. 10.1086/323201 PMC2692641 11550118

[15] de Truchis P, Lê MP, Daou M, Madougou B, Nouhou Y, Moussa Saley S, et al High efficacy of first-line ART in a West African cohort, assessed by dried blood spot virological and pharmacological measurements. J Antimicrob Chemother. 2016;71: 3222–3227. 10.1093/jac/dkw286 27439522

[16] Pozniak AL, Miller R, Ormerod LP. The treatment of tuberculosis in HIV-infected persons. ResearchGate. 1999;13: 435–45. 10.1097/00002030-199903110-00001 10197371

[17] Burman WJ, Gallicano K, Peloquin C. Therapeutic implications of drug interactions in the treatment of human immunodeficiency virus-related tuberculosis. Clin Infect Dis Off Publ Infect Dis Soc Am. 1999;28: 419–429; quiz 430. 10.1086/515174 10194057

[18] Branch RA, Adedoyin A, Frye RF, Wilson JW, Romkes M. In vivo modulation of CYP enzymes by quinidine and rifampin. Clin Pharmacol Ther. 2000;68: 401–411. 10.1067/mcp.2000.110561 11061580

[19] Cohen K, Meintjes G. Management of individuals requiring antiretroviral therapy and TB treatment. Curr Opin HIV AIDS. 2010;5: 61–69. 20046149 10.1097/COH.0b013e3283339309 PMC2936963

[20] Manosuthi W, Sungkanuparph S, Tantanathip P, Lueangniyomkul A, Mankatitham W, Prasithsirskul W, et al A randomized trial comparing plasma drug concentrations and efficacies between 2 nonnucleoside reverse-transcriptase inhibitor-based regimens in HIV-infected patients receiving rifampicin: the N2R Study. Clin Infect Dis Off Publ Infect Dis Soc Am. 2009;48: 1752–1759. 10.1086/599114 19438397

[21] Cohen K, van Cutsem G, Boulle A, McIlleron H, Goemaere E, Smith PJ, et al Effect of rifampicin-based antitubercular therapy on nevirapine plasma concentrations in South African adults with HIV-associated tuberculosis. J Antimicrob Chemother. 2008;61: 389–393. 10.1093/jac/dkm484 18096560

[22] Cupp-Vickery JR, Garcia C, Hofacre A, McGee-Estrada K. Ketoconazole-induced conformational changes in the active site of cytochrome P450eryF. J Mol Biol. 2001;311: 101–110. 10.1006/jmbi.2001.4803 11469860

[23] Erickson DA, Mather G, Trager WF, Levy RH, Keirns JJ. Characterization of the in vitro biotransformation of the HIV-1 reverse transcriptase inhibitor nevirapine by human hepatic cytochromes P-450. Drug Metab Dispos Biol Fate Chem. 1999;27: 1488–1495. 10570031

[24] Penzak SR, Kabuye G, Mugyenyi P, Mbamanya F, Natarajan V, Alfaro RM, et al Cytochrome P450 2B6 (CYP2B6) G516T influences nevirapine plasma concentrations in HIV-infected patients in Uganda. HIV Med. 2007;8: 86–91. 10.1111/j.1468-1293.2007.00432.x 17352764

[25] Ribera E, Pou L, Lopez RM, Crespo M, Falco V, Ocaña I, et al Pharmacokinetic interaction between nevirapine and rifampicin in HIV-infected patients with tuberculosis. J Acquir Immune Defic Syndr 1999. 2001;28: 450–453. 10.1097/00042560-200112150-00007 11744833

[26] Haas DW, Ribaudo HJ, Kim RB, Tierney C, Wilkinson GR, Gulick RM, et al Pharmacogenetics of efavirenz and central nervous system side effects: an Adult AIDS Clinical Trials Group study. AIDS Lond Engl. 2004;18: 2391–2400. 15622315

[27] Haas DW, Smeaton LM, Shafer RW, Robbins GK, Morse GD, Labbe L, et al Pharmacogenetics of long-term responses to antiretroviral regimens containing Efavirenz and/or Nelfinavir: an Adult Aids Clinical Trials Group Study. J Infect Dis. 2005;192: 1931–1942. 10.1086/497610 16267764

[28] Rotger M, Colombo S, Furrer H, Bleiber G, Buclin T, Lee BL, et al Influence of CYP2B6 polymorphism on plasma and intracellular concentrations and toxicity of efavirenz and nevirapine in HIV-infected patients. Pharmacogenet Genomics. 2005;15: 1–5. 15864119 10.1097/01213011-200501000-00001

[29] Wang J, Sönnerborg A, Rane A, Josephson F, Lundgren S, Ståhle L, et al Identification of a novel specific CYP2B6 allele in Africans causing impaired metabolism of the HIV drug efavirenz. Pharmacogenet Genomics. 2006;16: 191–198. 10.1097/01.fpc.0000189797.03845.90 16495778

[30] Rotger M, Tegude H, Colombo S, Cavassini M, Furrer H, Décosterd L, et al Predictive Value of Known and Novel Alleles of CYP2B6 for Efavirenz Plasma Concentrations in HIV-infected Individuals. Clin Pharmacol Ther. 2007;81: 557–566. 10.1038/sj.clpt.6100072 17235330

[31] Matimba A, Oluka MN, Ebeshi BU, Sayi J, Bolaji OO, Guantai AN, et al Establishment of a biobank and pharmacogenetics database of African populations. Eur J Hum Genet. 2008;16: 780–783. 10.1038/ejhg.2008.49 18382479

[32] Mehlotra RK, Bockarie MJ, Zimmerman PA. CYP2B6 983T>C polymorphism is prevalent in West Africa but absent in Papua New Guinea: implications for HIV/AIDS treatment. Br J Clin Pharmacol. 2007;64: 391–395. 10.1111/j.1365-2125.2007.02884.x 17391322 PMC2000644

[33] Dhoro M, Ngara B, Kadzirange G, Nhachi C, Masimirembwa C. Genetic Variants of Drug Metabolizing Enzymes and Drug Transporter (ABCB1) as Possible Biomarkers for Adverse Drug Reactions in an HIV/AIDS Cohort in Zimbabwe. Curr HIV Res. 2013;11: 481–490. 24517233 10.2174/1570162x113119990048

[34] Manicaland Centre for Public Health Research [Internet]. Available: http://www.manicalandhivproject.org/

[35] Gregson S, Garnett GP, Nyamukapa CA, Hallett TB, Lewis JJC, Mason PR, et al HIV decline associated with behavior change in eastern Zimbabwe. Science. 2006;311: 664–666. 10.1126/science.1121054 16456081

[36] Mei JV, Alexander JR, Adam BW, Hannon WH. Use of filter paper for the collection and analysis of human whole blood specimens. J Nutr. 2001;131: 1631S–6S. 11340130 10.1093/jn/131.5.1631S

[37] Edelbroek PM, van der Heijden J, Stolk LML. Dried blood spot methods in therapeutic drug monitoring: methods, assays, and pitfalls. Ther Drug Monit. 2009;31: 327–336. 10.1097/FTD.0b013e31819e91ce 19349929

[38] McDade TW, Williams S, Snodgrass JJ. What a drop can do: dried blood spots as a minimally invasive method for integrating biomarkers into population-based research. Demography. 2007;44: 899–925. 18232218 10.1353/dem.2007.0038

[39] ter Heine R, Rosing H, van Gorp E, Mulder J, Beijnen J, Huitema A. Quantitation of raltegravir in human plasma, dried blood spots and peripheral blood mononuclear cell lysate by means of LC-MS/MS. J Int AIDS Soc. 2008;11: P242.

[40] Schur N, Mylne A, Mushati P, Takaruza A, Ward H, Nyamukapa C, et al The effects of household wealth on HIV prevalence in Manicaland, Zimbabwe–a prospective household census and population-based open cohort study. J Int AIDS Soc. 2015;18. 10.7448/IAS.18.1.20063 PMC4655223 26593453

[41] Manzou R, Schumacher C, Gregson S. Temporal Dynamics of Religion as a Determinant of HIV Infection in East Zimbabwe: A Serial Cross-Sectional Analysis. PLOS ONE. 2014;9: e86060. 24465868 10.1371/journal.pone.0086060 PMC3896440

[42] Gregson S, Mushati P, Grusin H, Nhamo M, Schumacher C, Skovdal M, et al Social capital and women’s reduced vulnerability to HIV infection in rural Zimbabwe. Popul Dev Rev. 2011;37: 333–359. 22066129 10.1111/j.1728-4457.2011.00413.x PMC3302682

[43] Schaefer R, Gregson S, Takaruza A, Rhead R, Masoka T, Schur N, et al. Spatial Patterns of HIV Prevalence and Service Use in East Zimbabwe: Implications for Future Targeting of Interventions (Submitted). 2016; 10.7448/IAS.20.1.21409 PMC5467609 28364568

